# Distinct acute and delayed effects of temperature and atmospheric pressure on allergic rhinitis: a distributed lag non-linear time-series study in subtropical China

**DOI:** 10.3389/fpubh.2026.1874064

**Published:** 2026-07-31

**Authors:** Jian Peng, Ganglei Shen, Kehong Zhang, Jingui Wang

**Affiliations:** 1 Wuxi Longsha Medical School Research Institute, Wuxi Hospital Affiliated to Nanjing University of Chinese Medicine, Wuxi, China; 2 Department of Public Health, Wuxi Hospital Affiliated to Nanjing University of Chinese Medicine, Wuxi, China; 3 Office of the Hospital President, Wuxi Hospital Affiliated to Nanjing University of Chinese Medicine, Wuxi, China

**Keywords:** allergic rhinitis, atmospheric pressure, climate change, distributed lag non-linear model, temperature, time-series study

## Abstract

**Background:**

Meteorological conditions are increasingly recognized as important determinants of allergic diseases; however, their temporal dynamics and delayed effects on allergic rhinitis (AR) remain inadequately characterized, particularly in subtropical settings. This study aimed to examine the non-linear and lagged relationships between major meteorological variables and AR-related outpatient visits in Wuxi, a representative subtropical city in eastern China.

**Methods:**

A time-series analysis was conducted using data from 76,760 AR outpatient visits recorded in Wuxi, China, between November 2020 and December 2024. Daily meteorological variables, including mean temperature, atmospheric pressure, relative humidity, wind speed, and air pollution indicators were obtained from local monitoring stations. Distributed lag non-linear models (DLNM), coupled with quasi-Poisson regression, were employed to estimate exposure–response relationships and lagged effects, with adjustment for long-term trends, seasonality, and day-of-week effects.

**Results:**

AR outpatient visits showed clear seasonal variation, with a major autumn peak and a secondary spring peak, and were predominantly concentrated among children and younger adults. Daily mean temperature was positively and non-linearly associated with AR outpatient visits, with risk increasing progressively above approximately 15 °C. Compared with 15 °C, the cumulative relative risk reached 1.19 at 25 °C and 1.41 at 30 °C, and the association was mainly acute, peaking within lag 0–3 days. In the primary single-exposure DLNM, lower atmospheric pressure showed an inverse exposure–response pattern and a delayed lag structure, with the strongest association observed around lag 8–10 days. However, pressure-related estimates were less stable in co-meteorological-adjusted and age-stratified analyses, suggesting that low atmospheric pressure may reflect broader synoptic weather conditions rather than an isolated independent exposure. Relative humidity and wind speed showed no robust independent associations.

**Conclusion:**

In this humid subtropical population, higher temperature was consistently associated with rapid increases in AR outpatient visits. Lower atmospheric pressure showed a delayed association in the primary model, but this pattern was less robust after accounting for correlated meteorological variables and across age strata. These findings provide epidemiological evidence for climate-sensitive AR prevention and support the incorporation of meteorological indicators into seasonal surveillance, early warning systems, and healthcare resource planning.

## 1 Introduction

Allergic rhinitis (AR) is among the most prevalent chronic allergic disorders worldwide and constitutes a significant public health burden (1–3). It is characterized by nasal obstruction, rhinorrhea, sneezing, and itching, all of which can substantially impair sleep quality, daily functioning, academic performance, and work productivity (1, 2). Beyond its direct impact on quality of life, AR frequently co-occurs with asthma, conjunctivitis, and other allergic conditions, thereby increasing healthcare utilization and socioeconomic costs. Current estimates indicate that AR affects approximately 10–40% of the global population, with its prevalence continuing to rise in many regions (1, 3).

In recent years, growing attention has been paid to the impact of environmental change on allergic diseases. Climate change, rapid urbanization, shifts in land use, and increasing air pollution have substantially altered patterns of allergen exposure and respiratory health (4–7). Among these factors, meteorological conditions may play a particularly important role in both the onset and exacerbation of AR. Variables such as temperature, humidity, wind speed, and atmospheric pressure can influence pollen production, fungal spore release, allergen dispersion, and airway mucosal responses (5–7). For example, elevated temperatures may extend pollen seasons and enhance allergenicity, whereas unstable weather patterns may amplify short-term fluctuations in symptoms.

Despite increasing interest in this area, epidemiological evidence remains inconsistent. Previous studies have reported heterogeneous associations between meteorological variables and AR, with both the direction and magnitude of effects varying across regions and study designs (7–14). Much of the existing literature has focused on temperate or cold climates, where the health impacts of low-temperature exposure are often emphasized. In contrast, comparatively little is known about the effects of meteorological conditions typical of humid subtropical regions, such as high temperatures, abundant rainfall, and frequent low-pressure systems. These climatic features may create distinct allergen environments and disease responses that cannot be directly extrapolated from studies conducted in northern populations.

Wuxi, located in the Yangtze River Delta in eastern China, provides a valuable context for examining these relationships. The city has a typical subtropical monsoon climate, characterized by warm summers, relatively high humidity, abundant precipitation, and pronounced seasonal transitions. Furthermore, the surrounding Taihu Lake basin and dense network of waterways may further shape local atmospheric circulation and aeroallergen dynamics. Notably, AR outpatient visits in this region include a high proportion of pediatric cases, suggesting increased vulnerability among children. Nevertheless, region-specific evidence from such settings remains limited.

A further limitation of previous studies lies in their methodological approaches. Many analyses have used linear models or assumed same-day effects, whereas meteorological exposures often exert complex, non-linear, and delayed influences on health outcomes. For instance, both low and high temperatures may elevate disease risk, while the effects of weather changes may manifest several days after the initial exposure. Analytical strategies that do not account for such patterns may underestimate or misrepresent the true exposure–response relationship.

Distributed lag non-linear models (DLNM) provide a robust framework for simultaneously assessing non-linear associations and lagged effects over time. This approach has been widely applied in environmental epidemiology, particularly in studies of cardiovascular and respiratory outcomes, but has been less frequently utilized in research on AR, especially within subtropical populations (15–19).

Therefore, the present study aimed to investigate the associations between major meteorological factors and AR outpatient visits in Wuxi, China, using a time-series design combined with DLNM. Specifically, we sought to quantify the non-linear and lagged effects of temperature and atmospheric pressure, identify critical exposure windows, and generate region-specific evidence to inform climate-sensitive prevention strategies for AR.

## 2 Materials and methods

### 2.1 Study design and setting

We conducted an ecological time-series analysis to evaluate the short-term associations between meteorological factors and outpatient visits for AR in Wuxi. Time-series methods are widely employed in environmental epidemiology because they allow for the assessment of transient exposure effects on population-level health outcomes while accounting for long-term trends, seasonality, and day-specific temporal patterns.

Wuxi is situated in the Yangtze River Delta region of eastern China (31°07′–32°02′N, 119°31′–120°36′E) and experiences a humid subtropical monsoon climate. The city is characterized by hot summers, relatively mild winters, abundant annual precipitation, high ambient humidity, and well-defined seasonal transitions. Moreover, the presence of Taihu Lake and an extensive inland water network may influence local atmospheric circulation, moderate temperature fluctuations, facilitate moisture transport, and affect aeroallergen dispersion. These climatic and geographic features make Wuxi an appropriate setting for investigating meteorology-related patterns of allergic disease.

According to official geographical information from the Wuxi municipal government, the city is dominated by plains, with scattered low mountains and residual hills; the southern part consists mainly of water-network plains, the northern part of high sandy plains, and the central part of reclaimed low-lying water-network polders. The highest peak in the broader Wuxi administrative region is Huangtading in Yixing, with an elevation of 611.5 m, whereas the highest peak in the urban area is Sanmaofeng of Huishan, with an elevation of 328.98 m. Topographic data further indicate that the average elevation of Wuxi City is approximately 8 m, with most urban areas representing a low-altitude plain environment.

### 2.2 Health outcome data

Daily outpatient records were obtained from the Wuxi Center for Disease Control and Prevention for the period 2 November 2020 to 31 December 2024. The surveillance database comprises routine clinical visit records reported by healthcare institutions within the municipal reporting network.

Cases of AR were identified according to the *International Classification of Diseases, Tenth Revision* (ICD-10), using diagnostic codes J30.1–J30.4, which correspond to major subtypes of AR. To enhance diagnostic specificity, records indicating infectious rhinitis, acute sinusitis, or other acute upper respiratory tract infections were excluded where applicable.

The extracted variables included:

- Date of outpatient visit
- Sex
- Age
- Diagnostic category

Only patients residing in the urban districts of Wuxi were included in the primary analysis to enhance spatial comparability between health outcomes and meteorological exposure measurements. After quality control and eligibility screening, a total of 76,760 AR outpatient visits were retained.

For modelling purposes, individual visit records were aggregated into daily counts, which were subsequently used as the dependent variable in the time-series analyses.

### 2.3 Meteorological exposure data

Daily meteorological data were obtained from the Wuxi Meteorological Bureau. Measurements were collected from standardized, fixed-site monitoring stations operating in accordance with national meteorological quality-control protocols.

The primary meteorological variables included:

- Daily mean temperature (°C)
- Daily mean atmospheric pressure (hPa)
- Daily mean relative humidity (%)
- Daily mean wind speed (m/s)

These variables were selected based on prior evidence suggesting their potential relevance to allergic disease occurrence through mechanisms such as allergen generation, pollen dispersion, airway irritation, mucosal hydration, and synoptic weather transitions.

Meteorological observations were matched with daily AR visit counts according to calendar date, resulting in a unified analytical dataset with one observation per study day.

To avoid ambiguity in variable definition, the temperature variable used in the primary analysis was daily mean temperature, not daily maximum or minimum temperature. Atmospheric pressure and relative humidity were also defined as daily mean values. All meteorological variables were matched to AR outpatient visits by calendar date. The plausibility of the meteorological data was further checked by examining their observed ranges and descriptive statistics before formal modelling.

Daily air quality data for Wuxi during the study period were obtained from publicly available air quality monitoring records. The available indicators included the air quality index (AQI), PM2.5, PM10, SO2, CO, NO2, and 8-h ozone (O3_8h). These data were matched with daily AR outpatient counts and meteorological observations by calendar date.

Because PM2.5 and O3_8h are commonly used indicators of particulate and photochemical air pollution and were available throughout the study period, they were included in air-pollution-adjusted sensitivity models. PM10 was additionally considered in supplementary analyses. Air pollutants were modeled as smooth covariates to evaluate whether the main meteorological associations were robust after adjustment for co-occurring air pollution.

Although daily air quality data for Wuxi were publicly available and obtained for the entire study period, PM2.5 and O3_8h were analyzed exclusively in sensitivity models rather than being included in the primary single-exposure or co-meteorological-adjusted models. This approach was adopted for the following reasons: (1) the primary objective of this study was to characterize the non-linear and lagged effects of meteorological variables (particularly temperature and atmospheric pressure) in a humid subtropical monsoon climate; (2) meteorological factors and air pollutants are often strongly inter-correlated (e.g., temperature inversions and low-pressure systems influence pollutant accumulation and dispersion), which could introduce multicollinearity and compromise the stability and interpretability of the cross-basis functions for the main exposures; and (3) to maintain model parsimony while still rigorously evaluating robustness. PM2.5 and O3_8h were therefore included as smooth covariates in dedicated air-pollution-adjusted sensitivity models, with PM10 additionally examined in supplementary analyses.

### 2.4 Data cleaning and preprocessing

Prior to formal analysis, both the health and meteorological datasets underwent standardized preprocessing procedures.

For outpatient data:

- Duplicate entries were removed where identified
- Invalid dates were excluded
- Implausible age values were screened, and records were corrected or excluded where necessary.

For meteorological data:

- Internal range checks were conducted
- Obvious recording anomalies were reviewed
- Temporal continuity was verified across the full study period

The proportion of missing meteorological observations was minimal; therefore, a complete-case analysis was performed, and no multiple imputation procedures were required.

In addition, daily counts were visually inspected to identify abrupt spikes or discontinuities that could indicate reporting artifacts. No major structural anomalies requiring exclusion were detected.

### 2.5 Statistical framework

Because the outcome variable comprised daily counts and exhibited over dispersion relative to a standard Poisson distribution, generalized linear models with a quasi-Poisson link function were employed.

To simultaneously model:

1. Non-linear exposure–response relationships, and
2. Delayed lagged effects over time,

We applied DLNM (15–19). This framework is particularly well suited for environmental exposure research, as meteorological variables may exert both immediate and delayed health effects, and these associations are often non-linear across the full exposure range.

Exploratory Pearson correlation analyses were conducted to assess the unadjusted associations between daily AR outpatient visits and meteorological variables. Correlation coefficients, corresponding *p* values, and significance levels were reported.

### 2.6 Construction of cross-basis functions

For each meteorological exposure, a separate cross-basis matrix was constructed. This strategy was adopted to mitigate instability arising from collinearity among weather variables and to facilitate clearer interpretation of exposure-specific lag structures.

Natural cubic spline functions were applied to both the exposure and lag dimensions.

Based on prior literature, model parsimony, residual diagnostics, and interpretability considerations, the following parameterization was selected:

- 7 degrees of freedom (df) per year for calendar time to control long-term trends and seasonality
- 4 df for the exposure-response dimension
- 4 df for the lag dimension

The maximum lag period was set at 14 days, as allergic symptoms may occur immediately after exposure or may develop several days later due to cumulative inflammatory responses or delayed healthcare-seeking behavior.

### 2.7 Main model specification

The model can be expressed as:

log[E(Yt)]=α+cb(Xt,l)+ns(time,7×years)+DOWt

Where Yt represents the number of AR outpatient visits on day t, cb (Xt,l) denotes the cross-basis matrix describing the exposure–lag association of the meteorological variable, ns(time)controls for long-term and seasonal trends, and DOWt represents day of the week.

Relative risks (RRs) and corresponding 95% confidence intervals (95% CIs) were estimated using the median value of each meteorological variable as the reference level.

### 2.8 Control of confounding

Several temporal confounders were considered in model construction.

#### 2.8.1 Long-term trend and seasonality

Long-term temporal variations, including healthcare utilization trends, secular changes in disease prevalence, and recurring seasonal patterns, were controlled for using a smooth spline function of calendar time.

#### 2.8.2 Day of the week

Healthcare attendance may vary systematically between weekdays and weekends; therefore, day of the week was included as a categorical covariate in the model.

#### 2.8.3 Correlated meteorological variables

Because meteorological variables often co-vary, each primary exposure was modeled separately in the main analysis. Prior to model specification, pairwise correlations were assessed to reduce the potential impact of multicollinearity.

### 2.9 Sensitivity analyses

Several sensitivity analyses were conducted to test robustness:

- Varying calendar-time df from 6 to 8 per year
- Modifying exposure and lag spline complexity
- Changing maximum lag periods to 7, 14, and 21 days
- Comparing alternative reference values
- Performing calendar-period stratified analyses to account for potential changes in outpatient attendance during the COVID-19 pandemic
- Performing co-meteorological-adjusted models to evaluate the influence of correlated meteorological variables
- Performing air-pollution-adjusted models to assess potential confounding by co-occurring air pollution
- Conducting age-stratified analyses across major age groups.

To evaluate the possible influence of pandemic-related control measures and changes in healthcare-seeking behavior, the study period was divided into three calendar periods: a pandemic-control period from 2 November 2020 to 30 November 2022, a transition period from 1 December 2022 to 28 February 2023, and a post-adjustment period from 1 March 2023 to 31 December 2024. The transition period was considered separately because outpatient attendance and respiratory symptom profiles may have changed rapidly during this phase. Daily AR outpatient counts were first compared across calendar periods to describe differences in healthcare utilization. The main DLNM analyses for daily mean temperature and atmospheric pressure were then repeated separately for the pandemic-control and post-adjustment periods.

To address correlations among meteorological variables, co-meteorological-adjusted models were further fitted. When daily mean temperature was the primary exposure, daily mean atmospheric pressure, relative humidity, and wind speed were additionally adjusted for. When daily mean atmospheric pressure was the primary exposure, daily mean temperature, relative humidity, and wind speed were additionally adjusted for. These models were used as sensitivity analyses rather than as the primary models because of potential collinearity between meteorological variables.

Air-pollution-adjusted sensitivity models were also constructed. PM2.5 and O3_8h were included as smooth covariates in the primary air-pollution-adjusted models, and PM10 was additionally considered in supplementary analyses. These models were used to evaluate whether the main meteorological associations were robust after adjustment for co-occurring particulate and photochemical air pollution.

Age-stratified sensitivity analyses were conducted using four major age groups: 0–14 years, 15–44 years, 45–64 years, and ≥65 years. These analyses were performed to examine whether the main exposure–response patterns differed across major age strata.

In addition, the exposure–response curve shapes for daily mean temperature and atmospheric pressure were re-evaluated during sensitivity analyses. This re-evaluation included inspection of the observed exposure distributions, comparison of alternative reference values, and assessment of curve stability under different spline specifications and maximum lag periods. Particular attention was paid to whether the observed curves supported J-shaped, U-shaped, or monotonic exposure–response patterns.

The preliminary exposure–response curves were compared with the final model estimates. When curve interpretation differed across model specifications, the final interpretation was based on the model with better stability, biological plausibility, and consistency across sensitivity analyses. The preliminary curve outputs were retained and are provided in the Supplementary Materials for comparison.

### 2.10 Statistical software

All analyses were performed using R software (version 4.2.3; R Foundation for Statistical Computing, Vienna, Austria), primarily using the dlnm and splines packages.

All statistical tests were two-sided, and a *p* value < 0.05 was considered statistically significant.

**Figure 1:**
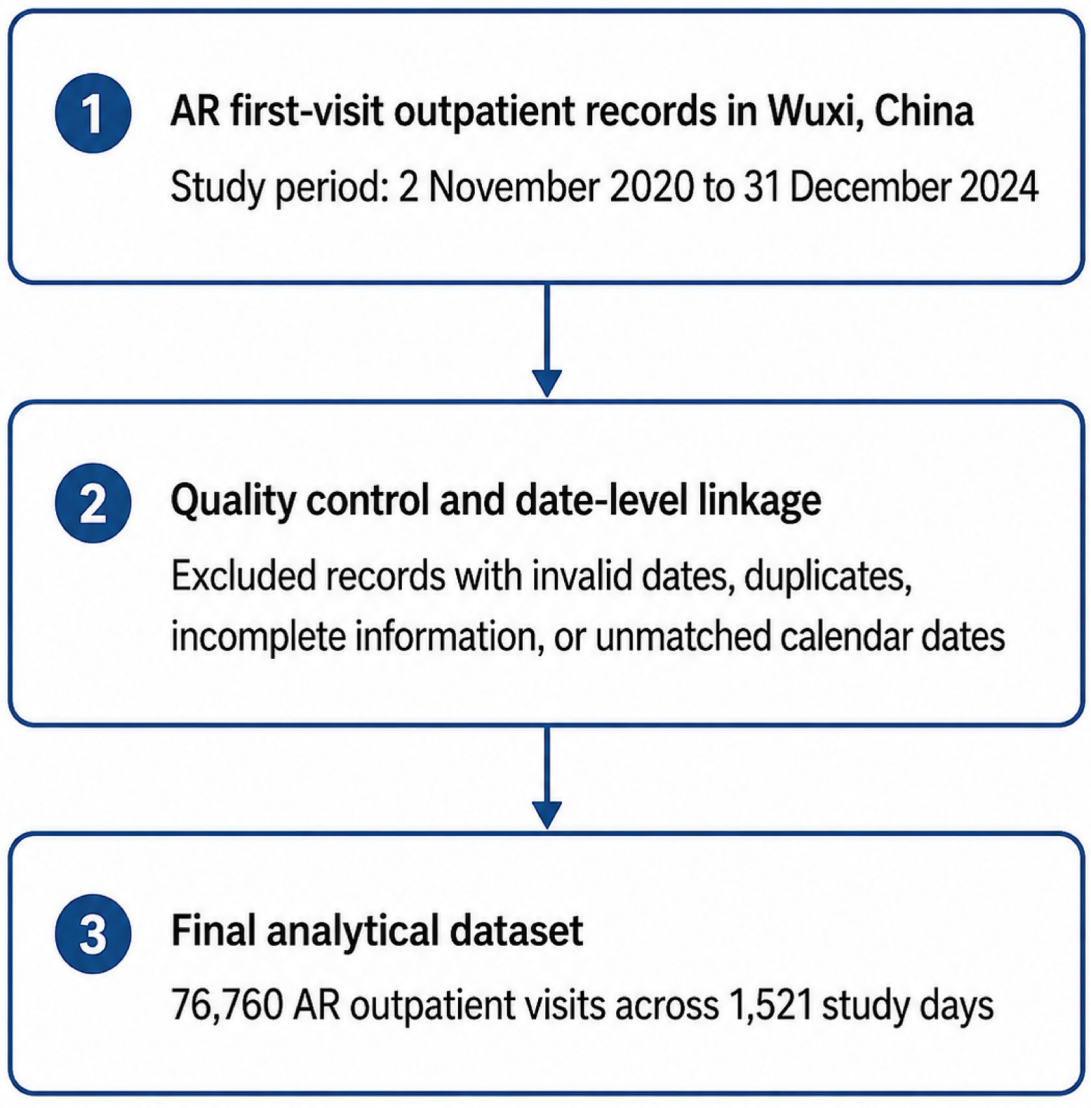
Study flowchart. Flowchart of case selection, quality control, and date-level linkage for the present time-series analysis. Allergic rhinitis (AR) first-visit outpatient records in Wuxi, China, from 2 November 2020 to 31 December 2024 were screened for eligibility. After quality control and exclusion of records with invalid dates, duplicate entries, incomplete information, or unmatched calendar dates, 76,760 eligible AR outpatient visits were retained. These records were linked by calendar date with daily meteorological data, including daily mean temperature, daily mean atmospheric pressure, daily mean relative humidity, and daily mean wind speed, yielding the final analytical dataset of 76,760 visits across 1,521 study days.

The overall process of case selection, data cleaning, and linkage with meteorological data is shown in Figure 1.

## 3 Results

### 3.1 Overall characteristics of AR outpatient visits

From 2 November 2020 to 31 December 2024, a total of 76,760 eligible AR outpatient visits were included in the final analysis. The mean daily number of visits was 50.47 (SD: 30.91), ranging from 0 to 222 visits per day. The highest daily count was recorded on 19 May 2024, indicating substantial short-term variability in outpatient demand Table 1.

**Table 1:** Robustness of atmospheric-pressure-related estimates across primary and co-meteorological-adjusted DLNM models.

Analytical setting	Exposure contrast	RR	95% CI	Interpretation
Primary single-exposure DLNM	1,010 hPa vs. 1,015 hPa	1.18	1.08–1.29	Low atmospheric pressure showed an increased cumulative estimate in the primary model.
Primary single-exposure DLNM	1,005 hPa vs. 1,015 hPa	1.52	1.34–1.72	The increased estimate was more evident at lower pressure levels in the primary model.
Co-meteorological-adjusted DLNM	1,010 hPa vs. 1,015 hPa	0.95	0.89–1.01	The increased-risk pattern was not retained after adjustment for correlated meteorological variables.
Co-meteorological-adjusted DLNM	1,005 hPa vs. 1,015 hPa	0.91	0.80–1.03	The pressure-related association was attenuated and became statistically non-significant after co-meteorological adjustment.

RR, relative risk; CI, confidence interval; DLNM, distributed lag non-linear model. The primary single-exposure model estimated the association between daily mean atmospheric pressure and allergic rhinitis outpatient visits using 1,015 hPa as the reference value. In the co-meteorological-adjusted model, daily mean temperature, relative humidity, and wind speed were additionally adjusted for. All models were adjusted for long-term trend, seasonality, and day of the week. Because daily mean atmospheric pressure was strongly correlated with daily mean temperature, the pressure-related results should be interpreted as a primary-model signal and a potential marker of broader synoptic weather conditions rather than as evidence of an isolated independent pressure effect.

The time-series pattern revealed recurrent seasonal peaks superimposed on moderate interannual variability. No evident abrupt long-term discontinuities in reporting volume were observed, supporting the overall stability and consistency of the surveillance dataset.

**Figure 2:**
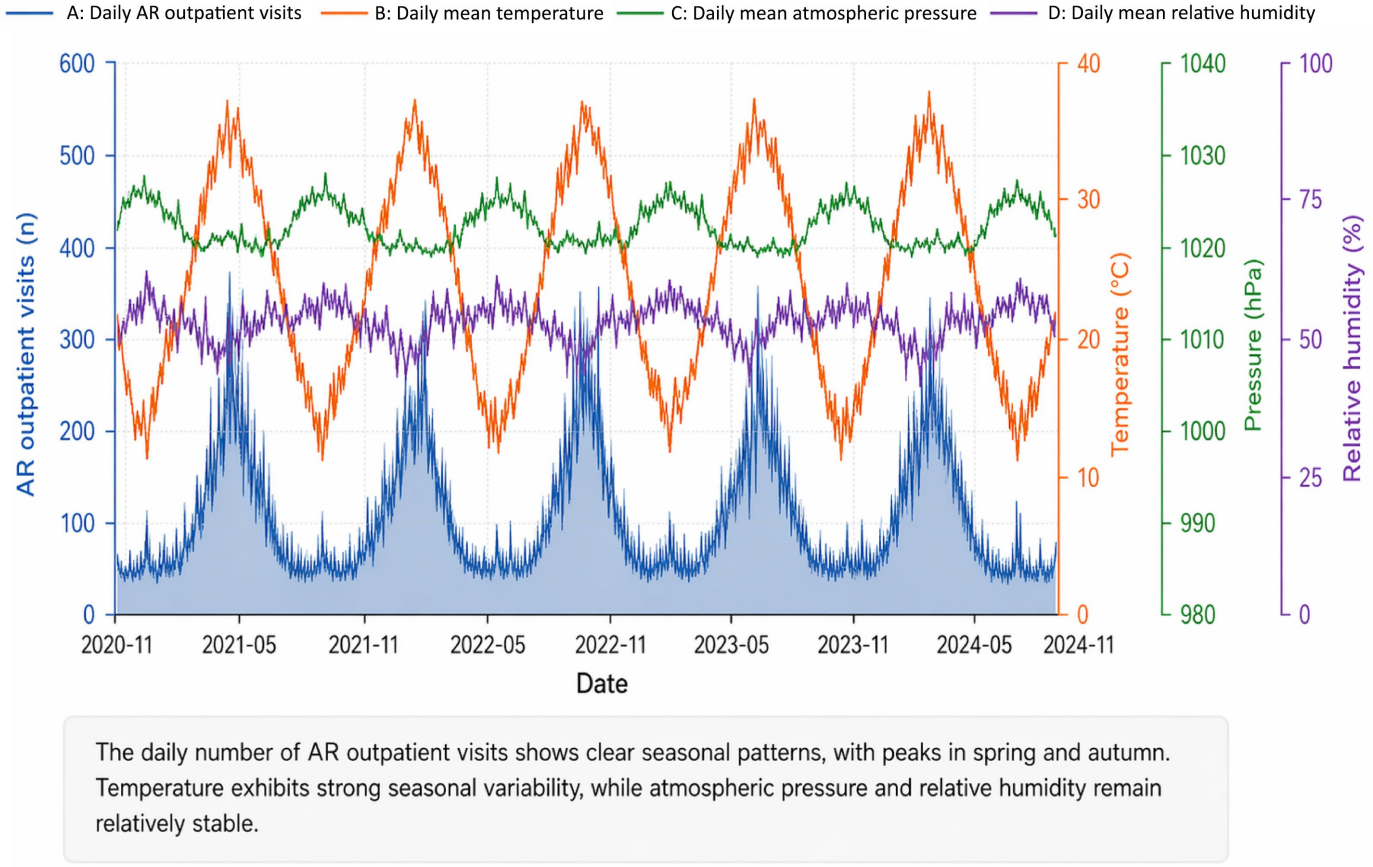
Daily time-series of AR outpatient visits and key meteorological variables. Daily time-series of allergic rhinitis (AR) outpatient visits and major meteorological variables in Wuxi, China, from 2 November 2020 to 31 December 2024. The four panels show: **(A)** Daily AR outpatient visits; **(B)** Daily mean temperature; **(C)** Daily mean atmospheric pressure; and **(D)** Daily mean relative humidity. Daily mean temperature, rather than daily maximum temperature, is presented. The figure demonstrates recurrent seasonal fluctuations in AR visits, marked annual cycles in temperature, inverse seasonal variation in atmospheric pressure, and short-term fluctuations in relative humidity.

The daily time-series patterns of AR outpatient visits and key meteorological variables are presented in Figure 2.

### 3.2 Meteorological characteristics and seasonal patterns

During the study period, the daily mean temperature averaged 17.64 °C, with a median of 17.8 °C and an observed range from −4.5 °C to 36.3 °C. The mean daily atmospheric pressure was 1016.20 hPa, ranging from 988.3 to 1042.5 hPa. The mean daily relative humidity was approximately 71.18%, ranging from 32.5 to 100%. These values were consistent with the humid subtropical monsoon climate of Wuxi and confirmed that daily mean temperature was used in the primary analysis.

**Figure 3:**
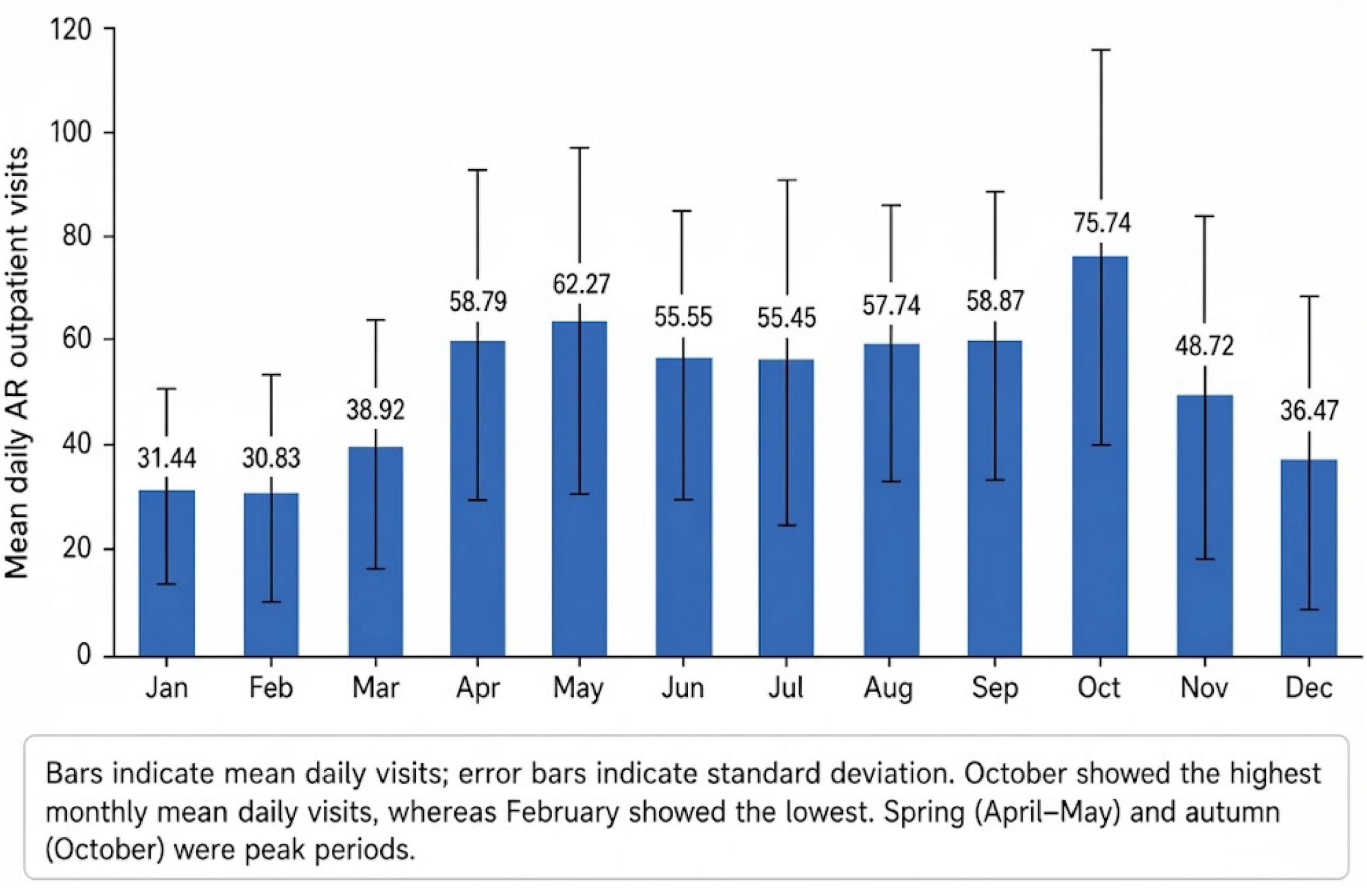
Monthly distribution of AR outpatient visits. Monthly mean daily numbers of allergic rhinitis (AR) outpatient visits in Wuxi during the study period. Bars represent monthly mean daily visits, error bars indicate standard deviations, and the line with markers shows the monthly trend. AR outpatient visits showed a clear bimodal seasonal pattern, with a major autumn peak in October and a secondary spring increase in April–May. The lowest monthly mean daily counts were observed in January and February. Seasonal differences were statistically significant according to ANOVA testing (*F* = 66.196, *p* < 0.001).

Monthly aggregation further confirmed the seasonal pattern observed in the daily time-series plot, as shown in Figure 3.

Pronounced seasonal variation was observed. The highest monthly mean outpatient count was recorded in October (75.74 visits/day), followed by elevated levels in May (62.27 visits/day) and September (58.87 visits/day). A secondary spring peak was observed in April (58.79 visits/day).

By contrast, winter represented the low-incidence period, especially:

- February: 30.83/day
- January: 31.44/day

Overall, AR visits followed a bimodal annual pattern characterized by:

- A spring increase
- A stronger autumn peak

Seasonal differences were statistically significant (ANOVA: *F* = 66.196, *p* < 0.001).

### 3.3 Demographic distribution

Among 75,696 records with valid age information, patients ranged in age from 0 to 96 years. Age information was missing or unavailable for 1,064 visits and was therefore excluded from age-stratified descriptive and sensitivity analyses.

Age-stratified analysis showed:

- 0–14 years: 34,211 visits
- 15–44 years: 29,612 visits
- 45–64 years: 9,696 visits
- ≥ 65 years: 2,177 visits.

Accordingly, the majority of visits occurred among individuals younger than 45 years.

**Figure 4:**
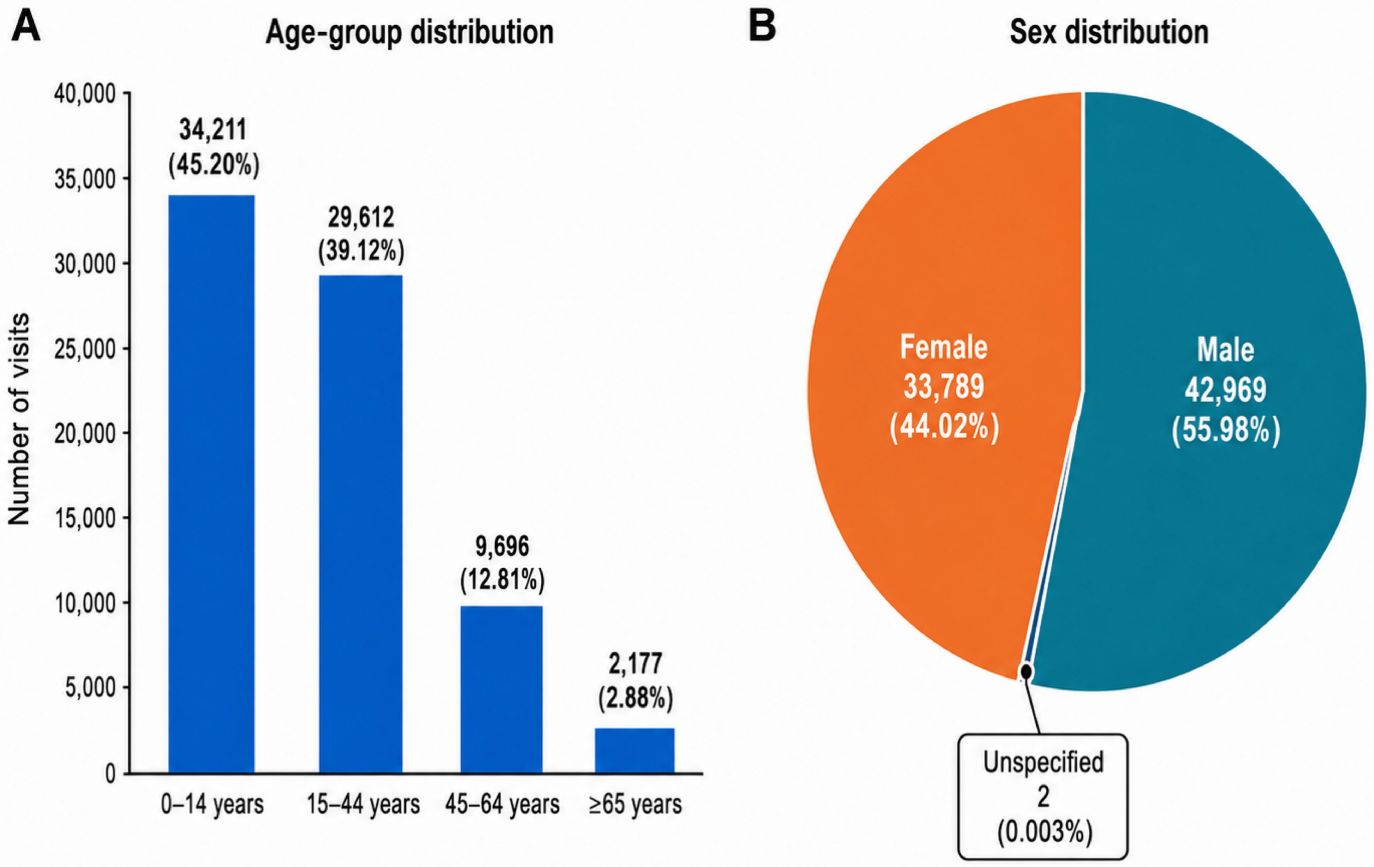
Age and sex distribution of AR outpatient visits. Demographic characteristics of AR outpatient visits in Wuxi during the study period. **(A)** Age-group distribution among records with valid age information, showing 34,211 visits among patients aged 0–14 years, 29,612 visits among those aged 15–44 years, 9,696 visits among those aged 45–64 years, and 2,177 visits among those aged ≥65 years. **(B)** Sex distribution of AR outpatient visits, showing 42,969 visits among male patients, 33,789 visits among female patients, and 2 visits with unspecified sex information.

Male patients accounted for 42,969 visits (55.98%), whereas female patients accounted for 33,789 visits (44.02%). Two records had unspecified sex information. The age-group and sex distributions of AR outpatient visits are shown in Figure 4.

### 3.4 Preliminary correlation analyses

Exploratory Pearson correlation analyses showed that temperature indicators were positively correlated with daily AR outpatient visits.

The strongest positive correlations were observed for:

- Daily maximum temperature (*r* = 0.312, *p* < 0.001)
- Daily mean temperature (*r* = 0.305, *p* < 0.001)
- Daily minimum temperature (*r* = 0.290, *p* < 0.001)

Mean vapor pressure was also positively correlated with outpatient visits (*r* = 0.261, *p* < 0.001), suggesting a potential role of warm, moist atmospheric conditions.

Atmospheric pressure indicators showed inverse associations:

- Maximum pressure (*r* = −0.253, *p* < 0.001)
- Mean pressure (*r* = −0.249, *p* < 0.001)
- Minimum pressure (*r* = −0.241, *p* < 0.001)

All reported correlations listed above were statistically significant at *p* < 0.001.

Other variables, including wind speed, rainfall, and sunshine duration, exhibited comparatively weak unadjusted correlations.

### 3.5 Non-linear and lagged effects of temperature

DLNM analyses suggested a non-linear increasing association between daily mean temperature and AR outpatient visits. The exposure–response curve did not support a true J-shaped pattern after re-evaluation; instead, the risk remained relatively stable at lower-to-moderate temperatures and increased progressively at higher temperatures, particularly above approximately 15 °C.

**Figure 5:**
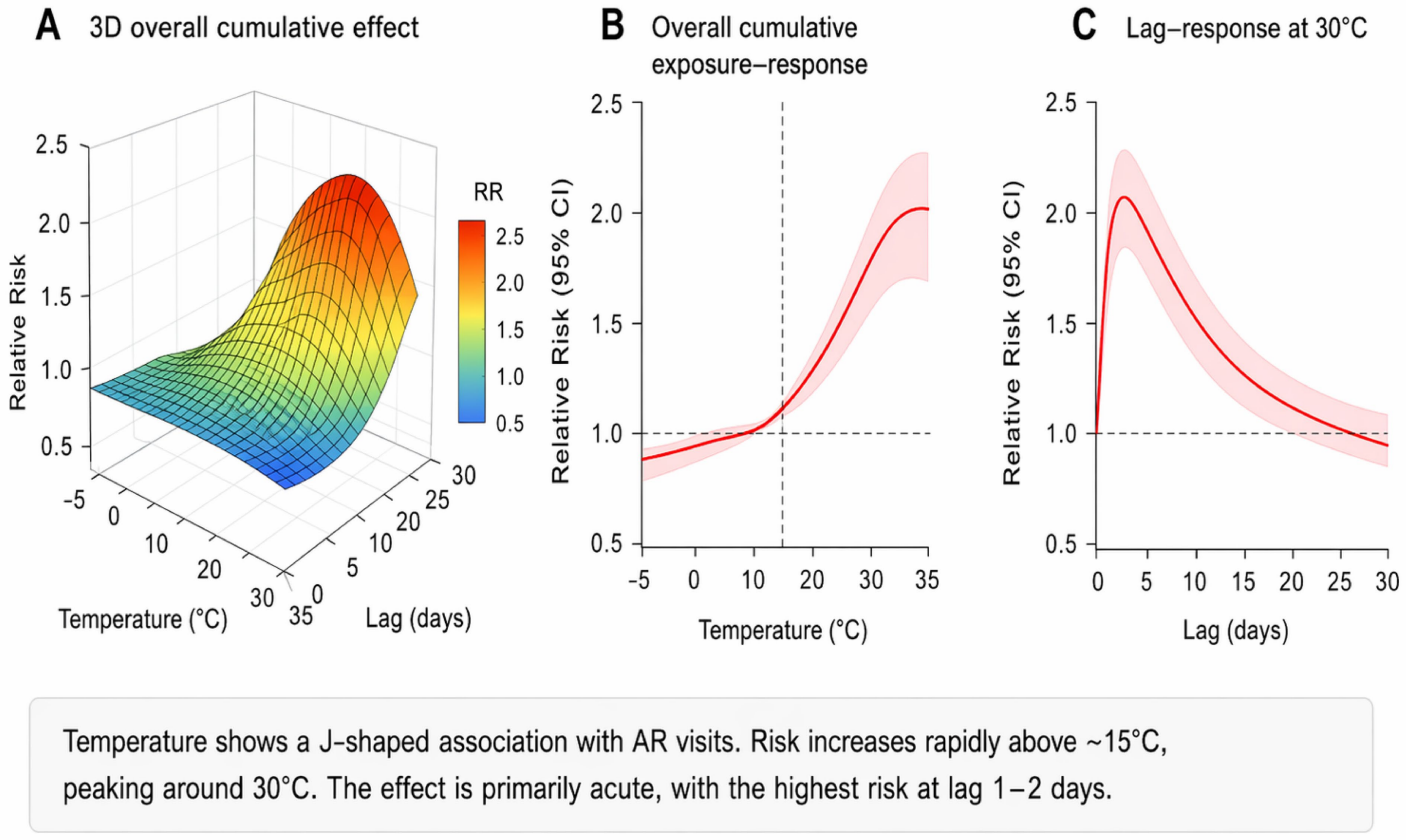
Associations between daily mean temperature and AR outpatient visits. Distributed lag non-linear model (DLNM) estimates for the association between daily mean temperature and allergic rhinitis (AR) outpatient visits. **(A)** Three-dimensional exposure–lag–response surface for daily mean temperature and AR risk. **(B)** Overall cumulative exposure–response curve for daily mean temperature, using 15 °C as the reference value. The curve indicates a non-linear increasing association, with AR risk rising progressively at higher temperatures, particularly above approximately 15 °C. **(C)** Lag-response association for high-temperature exposure at 30 °C, showing that the temperature effect was predominantly acute, with the strongest association occurring within lags of 0–3 days and peaking at approximately lag 1 day. Shaded areas indicate 95% confidence intervals.

The DLNM-derived exposure–lag–response associations for daily mean temperature are shown in Figure 5.

Using 15 °C as the reference level, the cumulative RR was 1.19 (95% CI: 1.10–1.29) at 25 °C and 1.41 (95% CI: 1.26–1.57) at 30 °C, indicating a higher AR risk under warmer conditions. The temperature effect was primarily acute, with the strongest association observed within lag 0–3 days and peaking at lag 1 day.

### 3.6 Non-linear and lagged effects of atmospheric pressure

**Figure 6:**
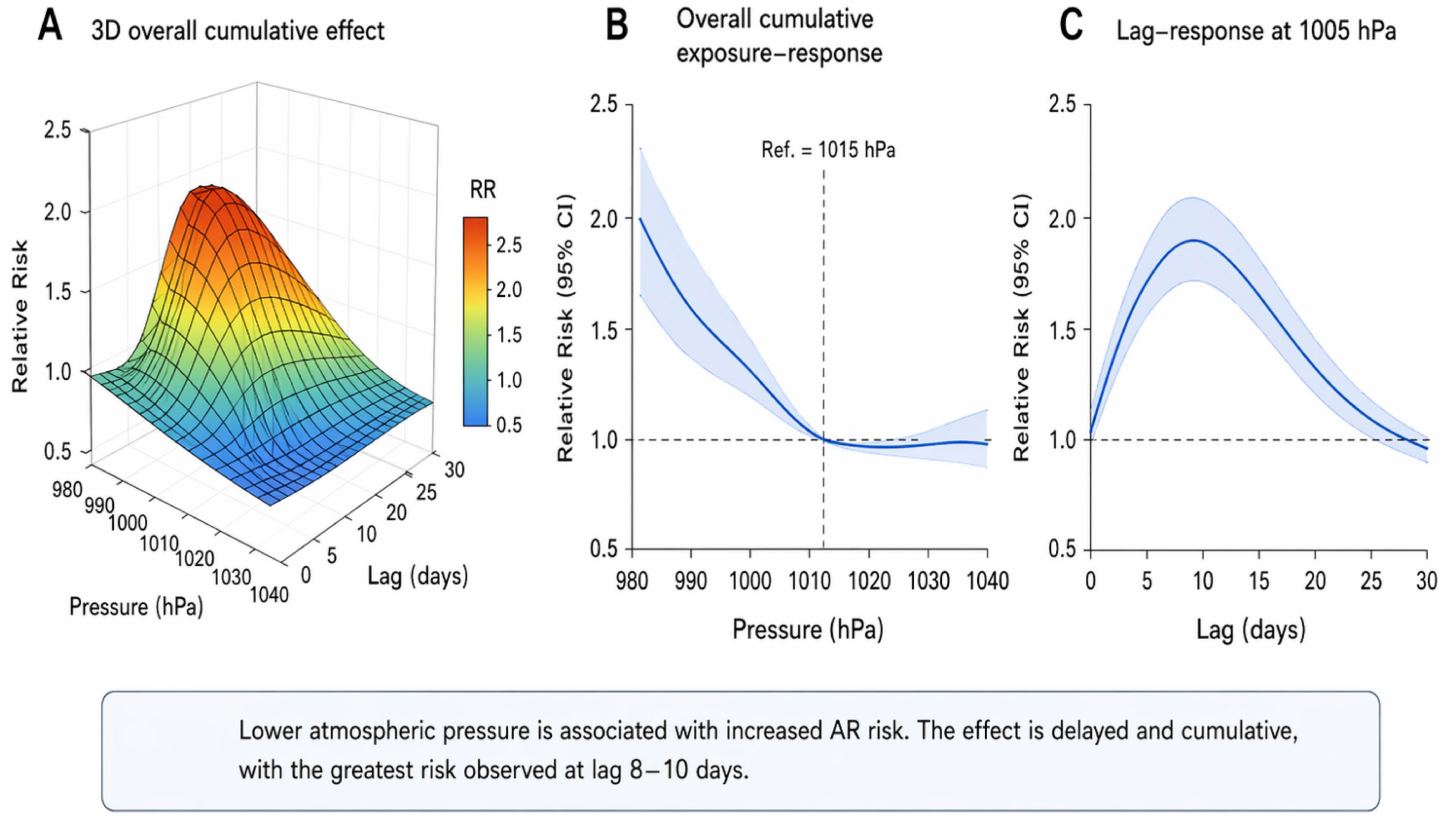
Associations between daily mean atmospheric pressure and AR outpatient visits in the primary single-exposure DLNM. Distributed lag non-linear model estimates for the association between daily mean atmospheric pressure and allergic rhinitis (AR) outpatient visits in the primary single-exposure model. **(A)** Three-dimensional exposure–lag–response surface for daily mean atmospheric pressure and AR risk. **(B)** Overall cumulative exposure–response curve for daily mean atmospheric pressure, using 1,015 hPa as the reference value. The curve shows an inverse exposure–response pattern in the primary model, with higher cumulative estimates at lower pressure levels, particularly below approximately 1,010 hPa. **(C)** Lag-response association for low-pressure exposure at 1005 hPa, showing a delayed pattern with the highest estimate observed at approximately lags of 8–10 days. Shaded areas indicate 95% confidence intervals. Because pressure-related estimates were less stable in co-meteorological-adjusted and age-stratified analyses, these findings should be interpreted as a primary-model signal rather than evidence of an isolated independent pressure effect.

In the primary single-exposure DLNM, daily mean atmospheric pressure showed an inverse non-linear association with AR outpatient visits. The DLNM-derived exposure–lag–response associations for daily mean atmospheric pressure are shown in Figure 6.

Using 1,015 hPa as the reference level, the primary model showed higher cumulative estimates at lower pressure levels. The cumulative RR was 1.18 (95% CI: 1.08–1.29) at 1010 hPa and 1.52 (95% CI: 1.34–1.72) at 1005 hPa. The association appeared more evident below approximately 1,010 hPa. The lag-response pattern suggested a delayed association, with the strongest estimate observed at approximately lag 8–10 days.

Because daily mean atmospheric pressure was strongly correlated with daily mean temperature, this pressure-related pattern was further evaluated in co-meteorological-adjusted and age-stratified sensitivity analyses. Therefore, the primary pressure findings should be interpreted as an exposure–response signal from the single-exposure model rather than definitive evidence of an independent pressure effect.

### 3.7 Calendar-period sensitivity analysis

Because the study period overlapped with the COVID-19 pandemic, calendar-period sensitivity analyses were further performed to evaluate whether changes in outpatient attendance could have influenced the main findings.

**Figure 7:**
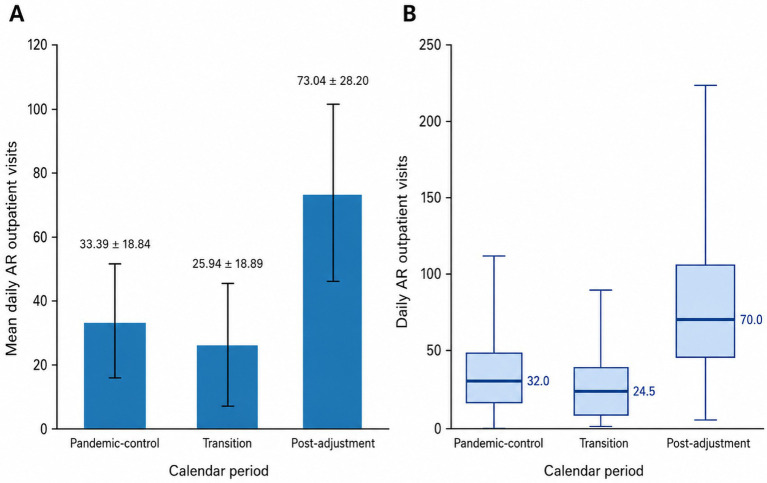
Calendar-period differences in AR outpatient visits. Daily allergic rhinitis (AR) outpatient visits were compared across three calendar periods: the pandemic-control period from 2 November 2020 to 30 November 2022, the transition period from 1 December 2022 to 28 February 2023, and the post-adjustment period from 1 March 2023 to 31 December 2024. Panel **(A)** shows the mean daily number of AR outpatient visits in each period, with error bars indicating standard deviations. Panel **(B)** shows the distribution of daily AR outpatient visits across the same periods, with boxes representing the interquartile range, horizontal lines representing medians, and whiskers representing the observed range. The post-adjustment period showed a substantially higher outpatient volume than the pandemic-control and transition periods, indicating clear calendar-period differences in baseline healthcare utilization.

The absolute number of AR outpatient visits differed across calendar periods, as shown in Figure 7. During the pandemic-control period from 2 November 2020 to 30 November 2022, 25,345 AR outpatient visits were recorded across 759 study days, with a mean daily count of 33.39 ± 18.84 and a median of 32.0 visits per day. During the transition period from 1 December 2022 to 28 February 2023, 2,335 visits were recorded across 90 days, with a mean daily count of 25.94 ± 18.89 and a median of 24.5 visits per day. During the post-adjustment period from 1 March 2023 to 31 December 2024, 49,080 visits were recorded across 672 study days, with a mean daily count of 73.04 ± 28.20 and a median of 70.0 visits per day.

After adjustment for month, day of the week, and meteorological variables, the daily number of AR visits in the post-adjustment period remained significantly higher than in the pandemic-control period (IRR = 2.13, 95% CI: 2.05–2.21, *p* < 0.001). This indicates substantial differences in baseline outpatient utilization across calendar periods.

**Figure 8:**
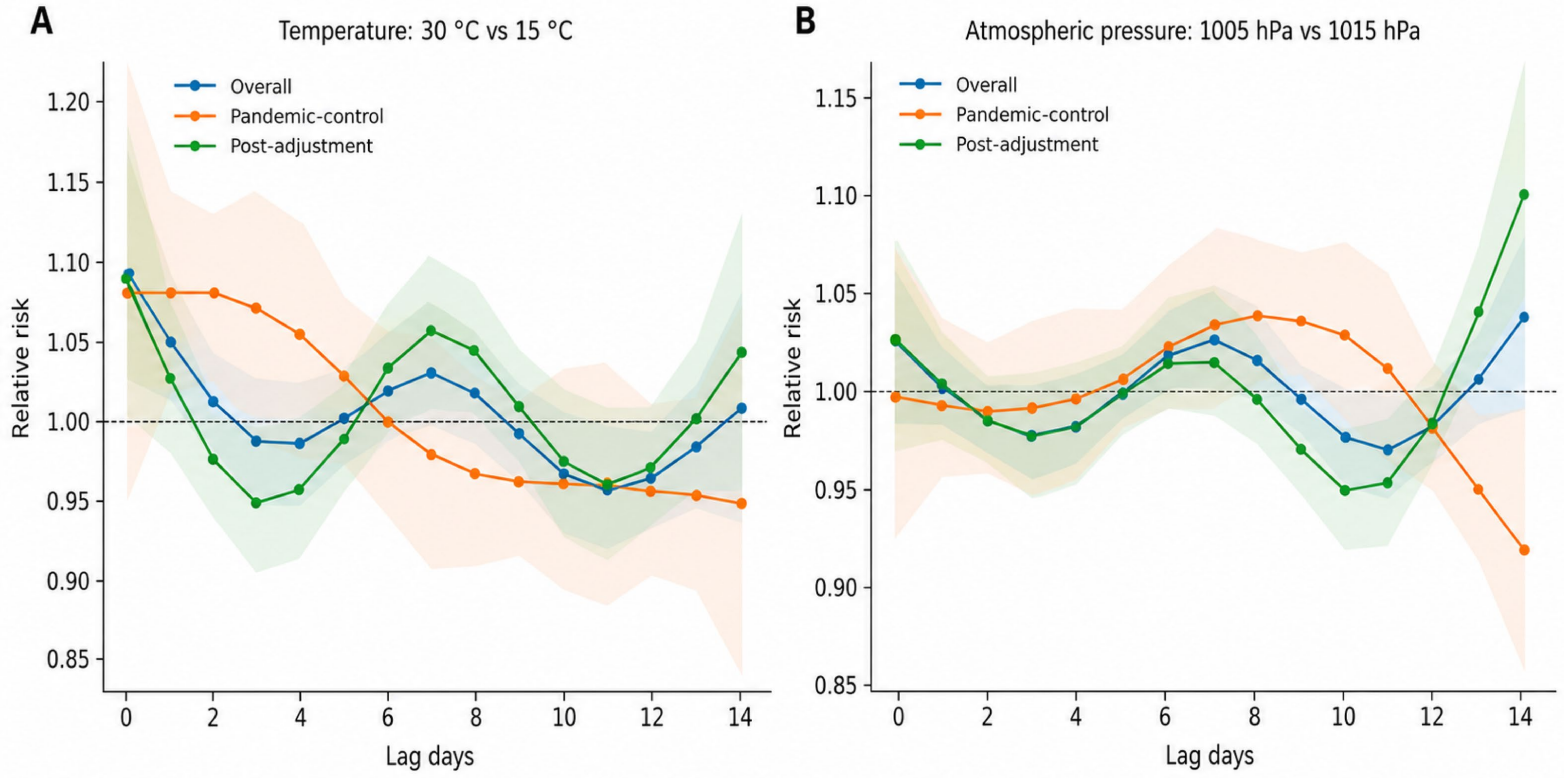
Period-stratified lag-response patterns for temperature and atmospheric pressure. Distributed lag non-linear model (DLNM) estimates were stratified by calendar period to assess the robustness of the main exposure–lag patterns. Panel **(A)** shows the lag-response associations for high-temperature exposure, comparing 30 °C with the reference temperature of 15 °C. Panel **(B)** shows the lag-response associations for low atmospheric pressure, comparing 1,005 hPa with the reference pressure of 1,015 hPa. Curves are shown for the overall study period, the pandemic-control period, and the post-adjustment period. The dashed horizontal line indicates a relative risk of 1.0, and shaded areas indicate 95% confidence intervals. Although the absolute outpatient volume differed across calendar periods, the major lag-response patterns for temperature and atmospheric pressure were broadly consistent.

The main DLNM analyses were then repeated separately for the pandemic-control and post-adjustment periods. As shown in Figure 8, although the absolute outpatient volume differed markedly between periods, the principal exposure–response patterns were broadly consistent. For temperature, the cumulative risk remained relatively stable at lower levels and increased progressively at higher temperatures in both periods, supporting a predominantly positive high-temperature association. For atmospheric pressure, lower pressure levels were consistently associated with increased cumulative risk in both periods, with the effect becoming more evident below approximately 1,010 hPa. Overall, the period-stratified analyses suggested that COVID-19-related factors may have affected the baseline level of AR outpatient visits, but did not materially alter the main meteorological exposure–response patterns observed in the overall analysis.

### 3.8 Sensitivity analysis for correlated meteorological variables

Because meteorological variables may be correlated with each other, we further examined pairwise correlations among the main meteorological indicators and performed co-meteorological-adjusted sensitivity analyses.

Daily mean temperature was strongly and inversely correlated with daily mean atmospheric pressure (*r* = −0.892), indicating substantial co-variation between these two variables. By contrast, the correlations among the remaining meteorological variables were weak to modest. The correlation coefficients were 0.111 between mean temperature and relative humidity, 0.087 between mean temperature and wind speed, −0.235 between atmospheric pressure and relative humidity, −0.126 between atmospheric pressure and wind speed, and −0.081 between relative humidity and wind speed. The variance inflation factors were 5.21 for mean temperature, 5.52 for atmospheric pressure, 1.14 for relative humidity, and 1.04 for wind speed, suggesting moderate collinearity between temperature and atmospheric pressure but no marked collinearity for relative humidity or wind speed.

In co-meteorological-adjusted models, temperature-related estimates were only slightly attenuated. For example, the cumulative RR at 30 °C vs. 15 °C was 1.41 (95% CI: 1.26–1.57) in the primary model and remained broadly similar after further adjustment for other meteorological variables. The overall exposure–lag pattern for temperature remained similar, supporting the robustness of the high-temperature association.

For atmospheric pressure, the estimates were less stable after adjustment for co-occurring meteorological variables, particularly because of the strong inverse correlation between atmospheric pressure and temperature. In the co-meteorological-adjusted model, the cumulative RR for 1,005 hPa compared with 1,015 hPa was 0.91 (95% CI: 0.80–1.03), indicating that the increased-risk pattern observed in the primary single-exposure model was not retained after simultaneous adjustment for other meteorological variables. These findings suggest that low atmospheric pressure should be interpreted primarily as a marker of broader synoptic weather conditions involving temperature, humidity, wind, and air-mass changes rather than as an isolated independent exposure. The detailed co-meteorological-adjusted sensitivity results are presented in Supplementary Table S1.

### 3.9 Air-pollution-adjusted sensitivity analysis

Adjustment for PM2.5 and O3_8h produced only minimal changes to the exposure–response and lag-response patterns for both daily mean temperature and atmospheric pressure (Supplementary Table S1). The cumulative relative risk estimates and the timing of peak effects remained materially consistent with those from the primary and co-meteorological-adjusted models. These results indicate that the observed associations between meteorological variables and AR outpatient visits are robust and unlikely to be substantially explained by short-term variation in particulate matter or photochemical air pollution. However, because air-pollution adjustment does not resolve the strong collinearity between atmospheric pressure and temperature, the pressure-related findings should still be interpreted in conjunction with the co-meteorological-adjusted sensitivity analyses.

### 3.10 Age-stratified sensitivity analysis

For atmospheric pressure, the age-stratified estimates were less stable and did not show a consistent age-specific increased-risk pattern. Compared with 1,015 hPa, the cumulative RR at 1005 hPa was 0.95 (95% CI: 0.80–1.14) among patients aged 0–14 years, 0.87 (95% CI: 0.76–1.01) among those aged 15–44 years, 0.87 (95% CI: 0.72–1.06) among those aged 45–64 years, and 0.94 (95% CI: 0.65–1.35) among those aged ≥65 years. These findings suggest that the pressure-related association observed in the primary model was not consistently reproduced across age strata. Overall, the age-stratified analyses supported the robustness of the temperature-related association, whereas age-specific estimates for atmospheric pressure were less consistent.

## 4 Discussion

In this multi-year ecological time-series study comprising 76,760 outpatient visits for AR, we identified heterogeneous short-term associations between meteorological variability and AR healthcare utilization in a humid subtropical city in eastern China. The most consistent finding was that elevated temperature was associated with a rapid, short-lived increase in AR outpatient visits, with the strongest estimates observed within the first few days after exposure. In the primary single-exposure DLNM, lower atmospheric pressure showed a delayed association with AR outpatient visits, with the highest estimate observed approximately 8–10 days after exposure. However, pressure-related estimates were less stable after adjustment for correlated meteorological variables and across age strata. These findings suggest that atmospheric pressure may be better interpreted as a marker of broader synoptic weather conditions rather than as an isolated independent exposure.

### 4.1 Elevated temperature as an acute trigger of AR

The association between higher temperatures and increased AR outpatient visits is biologically plausible. Heat can promote pollen release and enhance allergenicity, while also potentially lowering the threshold for symptom expression through effects on airway mucosa. In our data, AR risk remained relatively stable at lower temperatures but rose progressively once daily mean temperature exceeded approximately 15 °C, reaching a cumulative relative risk of 1.41 at 30 °C compared with 15 °C. This pattern suggests that moderate-to-high temperatures, rather than cold exposure, represent the main temperature-related driver of AR visits in this humid subtropical setting.

Several mechanisms may explain these findings. Warmer conditions can accelerate plant flowering, extend pollen seasons, increase pollen abundance, and enhance the expression of allergenic proteins. In the Wuxi region, spring warming likely intensifies tree and grass pollen release, while sustained warmth in autumn may prolong weed pollen activity. These effects align with the bimodal seasonal peaks observed in our study. In addition, warm and humid conditions favor dust mite survival and mold growth, which may increase indoor allergen exposure. Heat can also directly affect upper airway physiology by increasing epithelial permeability, promoting oxidative stress, and inducing mucosal vasodilation, thereby heightening local inflammatory responses.

The strongest temperature effect occurred within lag 0–3 days and peaked at lag 1 day. This rapid time course supports mechanisms involving immediate pollen release, short-term behavioral changes, or acute mucosal responses rather than slower cumulative processes.

### 4.2 Delayed and cumulative effects of low atmospheric pressure

A notable finding was the delayed association between low atmospheric pressure and AR outpatient visits. Unlike temperature, its effects were not immediate but built up over several days, with the strongest estimate observed around lag 8–10 days. This temporal pattern suggests that atmospheric pressure may act less as a direct trigger and more as a marker of broader weather systems that influence allergen exposure and symptom development.

Low-pressure systems are typically accompanied by changes in wind, cloud cover, precipitation, and atmospheric turbulence. These conditions can redistribute pollen, cause pollen grains to rupture into smaller respirable particles after rainfall, and promote mold spore release in the following days. As a result, atmospheric pressure likely captures a combination of meteorological processes that are not fully reflected by temperature alone. Some evidence also suggests that barometric changes may influence autonomic regulation and mucosal congestion, although direct data in allergic rhinitis remain limited.

The delayed peak at lag 8–10 days may reflect several interacting factors. These include repeated allergen exposure following a low-pressure event, gradual amplification of inflammation, accumulation of symptoms before individuals seek care, and delayed healthcare-seeking behavior for non-urgent allergic complaints. Because AR symptoms are often initially self-managed, clinical attendance may occur several days after the environmental trigger.

### 4.3 Comparison with previous literature

Our findings are broadly consistent with studies reporting positive associations between warmer weather and allergic or respiratory morbidity. However, much of the existing literature has been conducted in temperate regions of Europe and North America, as well as northern China, where cold exposure, indoor heating, and winter air pollution represent dominant environmental challenges (8–14).

In contrast, Wuxi represents a humid subtropical setting with:

- Long warm seasons
- High ambient moisture
- Strong spring–autumn transitions
- Frequent pressure variability
- Dense vegetation and water systems

Under such conditions, the dominant meteorological drivers of AR may differ from those reported in colder climates. Our findings therefore contribute important geographic diversity to the literature and caution against direct extrapolation of results across climate zones.

Evidence regarding atmospheric pressure remains comparatively limited and inconsistent. Some studies have reported associations between low pressure and increased respiratory symptoms or emergency visits, whereas others have found null results. Variations in study design, outcome definitions, exposure assessment methods, and statistical approaches likely account for these discrepancies. By explicitly modelling delayed cumulative effects, our study provides additional evidence that pressure-related associations may only become apparent when appropriate lag structures are incorporated (22–25).

### 4.4 Air pollution and aeroallergen co-exposures

Air pollution can confound studies examining meteorological effects on allergic diseases. We obtained daily PM2.5 and O3_8h data from Wuxi’s public monitoring network and included them as smooth covariates in sensitivity analyses. Adjustment for these pollutants produced only minimal changes to the exposure–response and lag-response patterns for both temperature and atmospheric pressure. This suggests that the observed meteorological associations are unlikely to be fully explained by short-term variation in air pollution.

Nevertheless, residual confounding by specific pollutants or their interactions with weather systems cannot be ruled out entirely. In addition, continuous daily pollen monitoring data were not available for Wuxi during the study period. Because meteorological conditions can influence AR through effects on pollen production, dispersion, and allergenicity, the absence of pollen data represents a limitation. Future research that combines high-resolution meteorological, air pollution, and standardized pollen data within distributed lag non-linear models would allow a more complete assessment of the relative contributions of these environmental factors.

### 4.5 Seasonal peaks and local aeroallergen ecology

AR outpatient visits showed a clear bimodal seasonal pattern, with a pronounced peak in autumn and a secondary rise in spring. This seasonal pattern is clinically meaningful and likely reflects interactions between regional allergen calendars and meteorological conditions.

#### 4.5.1 Spring peak

Spring increases may be associated with:

- Tree pollen
- Grass pollen
- Rapid warming
- Outdoor activity resumption

#### 4.5.2 Autumn peak

The stronger autumn peak may reflect:

- Weed pollen
- Mold spores after summer humidity
- Post-summer weather instability
- Continued warm temperatures extending allergen exposure

This pattern differs from that reported in some northern cities, where spring predominance is more commonly observed. Therefore, local aeroallergen ecology should be taken into account when interpreting the seasonal burden of AR.

### 4.6 Younger age groups as the Main burdened population

Children aged 0–14 years accounted for the largest proportion of AR visits, and nearly 80% of visits occurred among individuals younger than 45 years. This age distribution has several important implications.

#### 4.6.1 Biological susceptibility

Children have developing immune systems and may be more susceptible to allergen sensitization. In addition, smaller airway dimensions and more reactive mucosal tissues may contribute to increased symptom expression (26).

#### 4.6.2 Exposure patterns

School attendance, outdoor play activities, and daily commuting may increase exposure to environmental allergens. In addition, classroom crowding and exposure to indoor dust may contribute to persistent symptoms.

#### 4.6.3 Healthcare-seeking behavior

Parents may also be more likely to seek medical care promptly for symptomatic children than adults are for themselves.

These findings suggest that pediatric populations should be prioritized in preventive strategies, particularly during meteorologically high-risk periods (2, 3, 20).

### 4.7 Implications for clinical practice and public health

Although AR is sometimes perceived as a mild condition, its cumulative burden is substantial. Symptoms can impair sleep quality, academic performance, workplace productivity, and overall quality of life. In addition, recurrent outpatient demand can place considerable strain on healthcare services during seasonal peaks.

Our findings support several practical applications.

#### 4.7.1 Forecast-based early warning systems

Meteorological forecasts could be integrated with local pollen counts and air pollution data to identify periods of elevated AR risk. For example:

- Sudden warm spells in spring
- Sustained hot periods
- Low-pressure episodes during autumn

#### 4.7.2 Healthcare resource allocation

Hospitals and outpatient clinics may anticipate surges in AR visits and adjust staffing levels, medication stock, and appointment capacity accordingly.

#### 4.7.3 Individual preventive actions

Patients with recurrent AR may benefit from:

- Earlier initiation of antihistamines or intranasal corticosteroids
- Reduced outdoor exposure during peak-risk days
- Mask use where appropriate
- Improved indoor cleaning and ventilation

#### 4.7.4 School-based prevention

Given that children represented a major affected group, schools may serve as effective settings for seasonal education and symptom monitoring.

### 4.8 Climate change context

Climate change is expected to influence temperature extremes, seasonal duration, precipitation patterns, and atmospheric circulation. These changes may alter pollen seasons, enhance allergen potency, and modify weather systems associated with AR triggers.

In subtropical cities such as Wuxi, continued warming may prolong allergen-active periods and increase the frequency of meteorological conditions associated with AR exacerbations. Therefore, adaptive surveillance systems and climate-sensitive public health planning are likely to become increasingly important (4–7, 21).

### 4.9 Strengths of this study

This study has several notable strengths.

First, the analysis was based on a large real-world dataset comprising 76,760 outpatient visits collected over multiple years, enhancing statistical power and temporal coverage.

Second, the use of standardized meteorological observations improved the reliability of exposure assessment.

Third, the DLNM framework enabled the simultaneous evaluation of:

- Non-linear exposure thresholds
- Immediate effects
- Delayed cumulative effects

#### 4.9.1 Fourth, the main findings remained consistent across a range of sensitivity analyses

### 4.10 Overall interpretation

Taken together, these findings demonstrate that meteorological influences on AR are dynamic, exposure-specific, and temporally heterogeneous in humid subtropical settings. Elevated temperature appears to act as a rapid trigger, whereas low atmospheric pressure exerts slower, cumulative effects. Recognizing these distinct temporal signatures may help clinicians, researchers, and public health systems move beyond static seasonal assumptions toward more precise, weather-responsive prevention strategies.

### 4.11 Limitations

This study has several limitations. As an ecological time-series analysis based on aggregated daily outpatient counts, we cannot establish causal relationships at the individual level. Meteorological exposure was also assessed using fixed-site monitoring stations, which may not accurately reflect personal exposure across different residential, occupational, or indoor settings.

Although daily air quality data were available and used in sensitivity analyses, we did not include PM2.5 and O3_8h in the primary models. This decision was made to keep the focus on meteorological effects and to reduce the risk of multicollinearity with temperature and atmospheric pressure. In addition, continuous daily pollen monitoring data were not available for Wuxi during the study period. Because meteorological conditions can affect allergic rhinitis partly through their influence on pollen, some residual confounding by aeroallergens is possible.

Outpatient visit data reflect both the occurrence of symptoms and healthcare-seeking behavior. Variations in access to care, self-medication practices, public holidays, and broader health policies may therefore have influenced the observed daily counts. Finally, although we performed age-stratified analyses, the relatively small number of visits among adults aged 65 years and older means that estimates for this group should be interpreted with caution.

Despite these limitations, the study benefits from a large real-world dataset covering more than 4 years, linkage with reliable meteorological and air quality records, and the use of distributed lag non-linear models that allow simultaneous assessment of non-linear and delayed effects. The main findings remained consistent across multiple sensitivity analyses, which strengthens confidence in the results.

## 5 Conclusion

In this multi-year time-series study conducted in a humid subtropical city in eastern China, daily mean temperature showed the most consistent association with AR outpatient visits. Higher temperature was associated with rapid, short-term increases in AR risk, with the strongest association observed within the first few days after exposure. Lower atmospheric pressure showed a delayed association in the primary single-exposure model; however, this pattern was less robust after adjustment for correlated meteorological variables and was not consistently reproduced across age strata. Therefore, low atmospheric pressure should be interpreted as a potential indicator of broader synoptic weather conditions rather than as an independent meteorological determinant.

Most visits occurred among children, with clear peaks in both spring and autumn. Prevention strategies should therefore give particular attention to pediatric populations during high-risk seasons. Integrating meteorological indicators into disease surveillance, seasonal forecasting, and early warning systems may enhance AR prevention and healthcare preparedness in the context of ongoing climate change. Future studies that integrate continuous pollen monitoring, air pollution data, and individual-level exposure assessments within distributed lag non-linear frameworks are warranted to further elucidate the mechanisms linking climate variability and allergic rhinitis.

## Data Availability

The raw data supporting the conclusions of this article will be made available by the authors, without undue reservation.
