# Supplementary material for: Distinct acute and delayed effects of temperature and atmospheric pressure on allergic rhinitis: a distributed lag non-linear time-series study in subtropical China
Source: Front Public Health. 2026 Jul 31;14:1874064. doi: 10.3389/fpubh.2026.1874064 (PMC13475059; doi:10.3389/fpubh.2026.1874064)
Supplement: Supplementary file 1 [file Data_Sheet_1.pdf]

Supplementary

Supplementary Table S1. Atmospheric-pressure-related estimates across overall and age-stratified analyses.

Analysis	Population / subgroup	Exposure contrast	RR	95% CI	Interpretation
Primary single-exposure DLNM	Overall population	1010 hPa vs 1015 hPa	1.18	1.08–1.29	Increased estimate observed in the primary single-exposure model.
Primary single-exposure DLNM	Overall population	1005 hPa vs 1015 hPa	1.52	1.34–1.72	Stronger increased estimate at lower pressure levels in the primary single-exposure model.
Co-meteorological-adjusted DLNM	Overall population	1010 hPa vs 1015 hPa	0.95	0.89–1.01	The increased-risk pattern was not retained after adjustment for correlated meteorological variables.
Co-meteorological-adjusted DLNM	Overall population	1005 hPa vs 1015 hPa	0.91	0.80–1.03	The primary pressure-related signal was attenuated and became statistically non-significant after co-meteorological adjustment.
Age-stratified DLNM	0–14 years	1005 hPa vs 1015 hPa	0.95	0.80–1.14	No consistent increased-risk pattern observed.
Age-stratified DLNM	15–44 years	1005 hPa vs 1015 hPa	0.87	0.76–1.01	No consistent increased-risk pattern observed.
Age-stratified DLNM	45–64 years	1005 hPa vs	0.87	0.72–1.06	No consistent increased-risk

Analysis	Population / subgroup	Exposure contrast	RR	95% CI	Interpretation
		1015 hPa			pattern observed.
Age-stratified DLNM	≥65 years	1005 hPa vs 1015 hPa	0.94	0.65–1.35	Estimate was imprecise due to the relatively small number of visits in this subgroup.

Supplementary Table S1 note.
RR, relative risk; CI, confidence interval; DLNM, distributed lag non-linear model. The reference pressure level was 1015 hPa. The primary single-exposure model suggested an inverse exposure–response pattern between atmospheric pressure and allergic rhinitis outpatient visits. However, this pattern was not retained in co-meteorological-adjusted models and was not consistently reproduced across age strata. These findings support a cautious interpretation that low atmospheric pressure may serve as a marker of broader synoptic weather conditions rather than representing an isolated independent meteorological exposure. All models were adjusted for long-term trend, seasonality, and day of the week.

Supplementary Table S2. Pairwise correlations and variance inflation factors among main meteorological variables.

Panel A. Pairwise Pearson correlation coefficients

Variable pair	Correlation coefficient
Daily mean temperature vs daily mean atmospheric pressure	-0.892
Daily mean temperature vs relative humidity	0.111
Daily mean temperature vs wind speed	0.087
Daily mean atmospheric pressure vs relative humidity	-0.235
Daily mean atmospheric pressure vs wind speed	-0.126
Relative humidity vs wind speed	-0.081

Panel B. Variance inflation factors

Variable	VIF
Daily mean temperature	5.21
Daily mean atmospheric pressure	5.52
Relative humidity	1.14
Wind speed	1.04

Supplementary Table S2 note.
VIF, variance inflation factor. Daily mean temperature and daily mean atmospheric pressure showed strong inverse correlation and moderate collinearity, whereas relative humidity and wind speed showed no marked collinearity. This correlation structure supports the use of co-meteorological-adjusted models as sensitivity analyses and justifies cautious interpretation of pressure-related estimates.

Supplementary Table S3. Air-pollution-adjusted sensitivity analysis for temperature and atmospheric pressure

Exposure	Exposure contrast	Primary single-exposure model RR (95% CI)	Air-pollution-adjusted model RR (95% CI)	Interpretation
Daily mean temperature	30 °C vs 15 °C	1.41 (1.26–1.57)	1.38 (1.23–1.54)	Temperature association remained robust and materially unchanged after additional adjustment for PM2.5 and O3_8h.
Daily mean atmospheric pressure	1005 hPa vs 1015 hPa	1.52 (1.34–1.72)	1.47 (1.29–1.67)	Pressure estimate showed only minimal change after air pollution adjustment; however, this should still be interpreted together with co-meteorological-adjusted results due to strong correlation with temperature.

Supplementary Table S3 note.

RR, relative risk; CI, confidence interval; DLNM, distributed lag non-linear model. PM2.5 and O3_8h were included as smooth covariates in the air-pollution-adjusted models. The exposure–response and lag-response patterns for both temperature and atmospheric pressure remained materially consistent after adjustment for air pollution, indicating that the observed meteorological associations are robust to short-term variation in particulate matter and ozone. However, air-pollution adjustment does not address the strong collinearity between atmospheric pressure and temperature. Therefore, pressure-related findings should be interpreted in conjunction with the co-meteorological-adjusted sensitivity analyses rather than as evidence of an independent effect.
